# The Synthetic Antimicrobial Peptide 19-2.5 Interacts with Heparanase and Heparan Sulfate in Murine and Human Sepsis

**DOI:** 10.1371/journal.pone.0143583

**Published:** 2015-11-23

**Authors:** Lukas Martin, Rebecca De Santis, Patrick Koczera, Nadine Simons, Hajo Haase, Lena Heinbockel, Klaus Brandenburg, Gernot Marx, Tobias Schuerholz

**Affiliations:** 1 Department of Intensive Care and Intermediate Care, University Hospital RWTH, Aachen, Germany; 2 Department of Food Chemistry and Toxicology, Berlin Institute of Technology, Berlin, Germany; 3 Division of Biophysics, Forschungszentrum Borstel, Borstel, Germany; University of Leicester, UNITED KINGDOM

## Abstract

Heparanase is an endo-β-glucuronidase that cleaves heparan sulfate side chains from their proteoglycans. Thereby, heparanase liberates highly potent circulating heparan sulfate-fragments (HS-fragments) and triggers the fatal and excessive inflammatory response in sepsis. As a potential anti-inflammatory agent for sepsis therapy, peptide 19–2.5 belongs to the class of synthetic anti-lipopolysaccharide peptides; however, its activity is not restricted to Gram-negative bacterial infection. We hypothesized that peptide 19–2.5 interacts with heparanase and/or HS, thereby reducing the levels of circulating HS-fragments in murine and human sepsis. Our data indicate that the treatment of septic mice with peptide 19–2.5 compared to untreated control animals lowers levels of plasma heparanase and circulating HS-fragments and reduces heparanase activity. Additionally, mRNA levels of heparanase in heart, liver, lung, kidney and spleen are downregulated in septic mice treated with peptide 19–2.5 compared to untreated control animals. In humans, plasma heparanase level and activity are elevated in septic shock. The *ex vivo* addition of peptide 19–2.5 to plasma of septic shock patients decreases heparanase activity but not heparanase level. Isothermal titration calorimetry revealed a strong exothermic reaction between peptide 19–2.5 and heparanase and HS-fragments. However, a saturation character has been identified only in the peptide 19–2.5 and HS interaction. In conclusion, the findings of our current study indicate that peptide 19–2.5 interacts with heparanase, which is elevated in murine and human sepsis and consecutively attenuates the generation of circulating HS-fragments in systemic inflammation. Thus, peptide 19–2.5 seems to be a potential anti-inflammatory agent in sepsis.

## Introduction

Sepsis is a common and life-threatening disease especially in medical and surgical intensive care patients with mortality rates up to 60% [1]. It is characterized by a systemic inflammatory response to infection triggered by both pathogen-associated molecular patterns (PAMPs) as well as endogenous danger-associated molecular patterns (DAMPs) [2,3]. Here, heparan sulfate and the enzyme heparanase play key roles. Heparan sulfates are linear polysaccharides composed of repeating disaccharide subunits, which are D-glucosamine and D-glucuronic acid in their unmodified form. They are attached on a cell surface bound core protein [4]. Heparanase is an endo-β-glucuronidase that cleaves the heparan sulfate side chains within highly sulfated regions. Thereby, heparanase liberates highly potent circulating heparan sulfate-fragments (HS-fragments) [5]. Circulating HS-fragments are known to act as highly potent DAMPs and trigger the pro-inflammatory response in sepsis through Toll-like receptor 4-dependet pathways [6,7]. Thus, new anti-inflammatory agents interacting with heparanase and reducing the levels of circulating HS-fragments may be promising candidates for sepsis therapy. The naturally occurring antimicrobial cathelicidin peptide LL-37 neutralizes the pro-inflammatory action of PAMPs and DAMPs [8], however its therapeutic use is limited due to intrinsic toxicity [9,10]. Therefore, the challenge is to develop synthetic peptide-based drugs on the basis of naturally occurring antimicrobial peptides without causing harm.

The synthetic antimicrobial peptide 19–2.5 belongs to the class of synthetic anti-lipopolysaccharide peptides (SALP = synthetic anti-LPS peptides). However its activity is not restricted to Gram-negative bacterial infection [11,12], as Peptide 19–2.5 shows anti-inflammatory activity against Gram-negative and Gram-positive bacteria as well as against viruses [13]. In this way, it limits systemic inflammation and protects mice from lethal septic shock [11,14]. We recently reported that peptide 19–2.5 is able to decrease the inflammatory response in murine cells stimulated with both PAMPs and DAMPs [3]. However, the interaction of peptide 19–2.5 and DAMPs is still unclear *in vivo*.

Thus, our study aimed to investigate peptide 19–2.5 interaction with heparanase and HS-fragments in murine and human sepsis. We investigated peptide 19–2.5 treatment in septic mice using cecal ligature and puncture (CLP), the gold-standard method for studying polymicrobial sepsis in mice [15]. Furthermore we used the plasma of patients with septic shock as well as of healthy humans and performed isothermal titration calorimetry (ITC) to study the thermodynamics of binding of peptide 19–2.5 with heparanase and HS-fragments.

The findings of our present study indicate that peptide 19–2.5 interacts with heparanase and consecutively attenuates the generation of circulating HS-fragments in systemic inflammation. Thus, peptide 19–2.5 may be a promising tool for sepsis therapy.

## Materials and Methods

### Plasma sampling

As described before [3], we used plasma from 18 adult individuals within 24 hours after presentation with septic shock, according to the ACCP/SCCM definition [16] after written informed consent of patients or their legal representative. Individuals below 18 years were excluded. Furthermore, we used plasma from healthy human donors after written informed consent (n = 10). No personal or identifying information was collected from study participants. All samples are stored in the RWTH centralized Biomaterial Database (RWTH cBMB) of the University Hospital RWTH Aachen. The local ethics committee (University Hospital RWTH Aachen, EK 206_09) approved this study before inclusion of the first individual.

### Heparanase and heparan sulfate ELISA

Heparanase levels in human and murine plasma were measured using a commercial ELISA kit (No.: E03H0100, AMS Biotechnology, Oxon, United Kingdom) according to the manufacturer’s instructions. The ELISA´s detection range is 1.0 ng/ml– 5000 ng/ml.

### Heparan sulfate ELISA

Levels of circulating HS-fragments in murine plasma were measured using a commercial ELISA kit (No.: E0623Ge, AMS Biotechnology, Oxon, United Kingdom) according to the manufacturer’s instructions. Circulating HS-fragments in plasma were defined by the sum of the HS derived disaccharides obtained after enzymatic cleavage by heparanase. Due to the ELISA´s detection range of 4.69 ng/ml– 300 ng/ml samples were diluted 1:30 in physiological saline (0.9%). The sensitivity of the ELISA is 1.17ng/ml. Control experiments revealed no detection of 20μg/ml of peptide 19–2.5 solved in physiological saline (0.9%) by the ELISA. Furthermore, adding peptide 19–2.5 to the positive control of the ELISA (300ng/ml heparan sulfate) did not alter the detected HS concentration.

### Heparanase activity assay

Heparanase activity in human and murine plasma was quantified using the commercial available Heparanase Assay Kit (No.: Ra001-BE-K, AMS Biotechnology, Oxon, United Kingdom) according to the manufacturer’s instructions.

### Animal model

All animal experiments have been performed in accordance with the guidelines of the Institutional Animal Care and Use Committee (IACUC) and the National Animal Welfare Law and after approval by the responsible government authority ("Landesamt für Natur, Umwelt und Verbraucherschutz": LANUV-NRW, Germany: AZ 8.87–50.10.35.09.044). All efforts were made to minimize suffering of the animals. Mice were housed under standard laboratory conditions (room temperature 21 ± 1°C; relative humidity 40–55%; photoperiod 12 light:12 dark) and supplied with a standard feed and tap water *ad libitum*. The animals were handled according to guidelines of the Federation of European Laboratory Animal Science Associatinon (FELASA). This study used humane endpoints according to references ot the Society of Laboratory animal science (GV-SOLAS).

The murine model of polymicrobial sepsis was divided into three steps as described before [14]. Briefly, in a first step 12 NMRI mice underwent a catheterization procedure under general (isoflurane 1% to 2% in oxygen/air mix with a FiO2 of 0.3) and local (0.2 ml lidocaine 2%) anesthesia. A central vein catheter (PE-tube, self-made) was implanted in the jugular vein. After further local infiltration with lidocaine, the neck was closed by single suture. To prevent hypothermia all animals were kept on a heating pad throughout the surgical procedure. Mice were transferred back into the cage to rest for 48h. The i.v.-line was connected to the syringe pump at a rate of 100 μl/h (NaCl 0.9%).

In a second step, general and local anesthesia for cecal ligature and puncture (CLP) was performed as described above. The animal were transferred to the cage and reconnected with the i.v.-line. Depending on the group, peptide 19–2.5 infusion (20 μg/ml, 100 μl/h) or NaCl 0.9% infusion (100 μl/h) was started. During preceding studies the dose of 20μg/ml of peptide 19–2.5 was found to be the most beneficial with least harm to the animals [3,11–14,17,18].

Finally, in a third step all animals were killed 24 h after CLP. Therefore, the animals were anesthetized as described above, brought to a prone position and the abdomen and thorax were opened. Blood was sampled in pre-citrated syringes. Directly after killing the animal under general anesthesia by cervical dislocation heart, liver, lung, kidney and spleen were snap-frozen in liquid hydrogen until further procedures.

### RNA extraction and PCR

As described before [14], total RNA was extracted from snap-frozen tissue (RNeasy Mini Kit, Qiagen, Hilden, Germany) and reverse transcribed to cDNA (Quanti Tec, Rev Transcription Kit, Qiagen, Hilden, Germany). cDNA was analyzed by quantitative real-time PCR performed with Power SYBR Green PCR Master Mix on a StepOnePlus (all Applied Biosystems, Foster City, CA, USA) using a mouse specific primer (biomers, Ulm, Germany) for heparanase 5’-TTTGCAGCTGGCTTTATGTG-3’ (for) and 5’-GTCTGGGCCTTTCACTCTTG-3’ (rev). Beta-actin was used as an endogenous normalization control: 5’-GCTCTTTTCCAGCCTTCCTT-3’ (for), 5’-CGG ATGTCAACGTCACACTT-3’ (rev). The following conditions were used: 95°C for 3 minutes; then, 40 cycles at 95°C for 30 seconds, 57°C for 30 seconds and 72°C for 30 seconds. Expression was normalized to the housekeeping gene ß-actin.

### Isothermal Titration Calorimetry

Isothermal titration calorimetry (ITC) experiments were performed as previously described [11]. Briefly, the binding of peptide 19–2.5 to heparanase or HS-fragments was recorded by measuring the enthalpy change of the reaction at 37°C. For this, a total of 100 μg/ml heparanase (R&D Systems Europe Ltd., Abingdon, United Kingdom) and 200 μg/ml HS-fragments (H7640 Sigma-Aldrich, St. Louis, MO, USA) were dispersed into the calorimetric cell, and 2 mM peptide 19–2.5 was titrated to this dispersion stepwise in 3 μl portions. After exploring experiments, the concentration of HS was 100 μg/ml titrated with 1.5 μl of peptide 19–2.5 (2 mM) 20 times at 37°C.

### Statistical analyses

All data are given as mean ± standard deviation (SD). As described recently [14], the PCR-derived mRNA expressions were analyzed using a relative expression software tool (REST, (http://www.gene-quantification.de/rest.html, rest-mcs-beta-9august 2006) performing a randomization test [19]. This tool avoids assumptions on distributions and applies a pair wise fixed reallocation randomization test, which reallocates control and sample groups (= pair wise fixed reallocation). The expression ratios are calculated on the basis of the mean crossing point (CP) values for reference and target genes [14,19]. We used a multiple t-test with Holm-Šídák correction when comparing differences between groups. A p-value of p < 0.05 was considered significant for all tests. We performed all calculation and figures using GraphPad Prism 6 (GraphPad, San Diego, CA, USA).

## Results and Discussion

### Patient characteristics

Patient and healthy human donor characteristics are shown in Table 1. All patients met criteria for septic shock according to the ACCP/SCCM definitions [16].

**Table 1:** Study population characteristics.

	Healthy (n = 10)	Septic shock (n = 18)
Age (years)	67 ± 19	70 ± 15
Male (%)	50%	78%
Focus of infection (%)		
Pulmonary		33%
Abdominal		33%
Miscellaneous		17%
Other		17%
White blood count (x 10^9^/l)		12.4 ± 5.7
C-reactive protein (mg/l)		170.2 ± 114.2
APACHE II score		16.8 ± 4.7
SOFA score		7.9 ± 2.7
90-day survival		47%

APACHE II score (Acute Physiology and Chronic Health Evaluation II score), SOFA (Sequential organ failure assessment score)

### Peptide 19–2.5 reduces heparanase level *in vivo*

Several studies indicate that heparanase plays a role in sepsis-associated pulmonary [20] and renal [21] failure. However, the measurements are limited to tissue levels in certain organs [20,21]. Our findings represent the first observations of *plasma* heparanase levels in murine and human sepsis.

**Fig 1:**
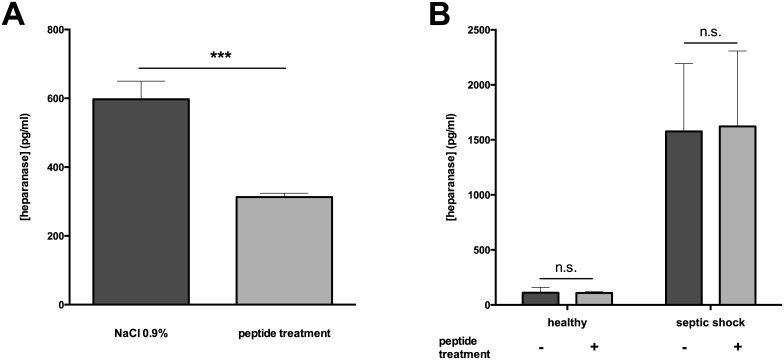
Heparanase level in murine (A) and human (B) sepsis. Plasma was obtained from mice subjected to cecal ligature and puncture (CLP) and treated with peptide 19–2.5 (20 μg/ml, n = 6) or NaCl 0.9% as a control (n = 6). Moreover, plasma samples were collected from control healthy volunteers (n = 10) and from patients with septic shock (n = 18). Peptide 19–2.5 (20 μg/ml) was added ex vivo to the plasma. Data are presented as mean ± SD. P-values represent the statistical differences between groups using a multiple t-test with Holm-Šídák correction. ***p < 0.0001, n.s. = non significant.

**Fig 2:**
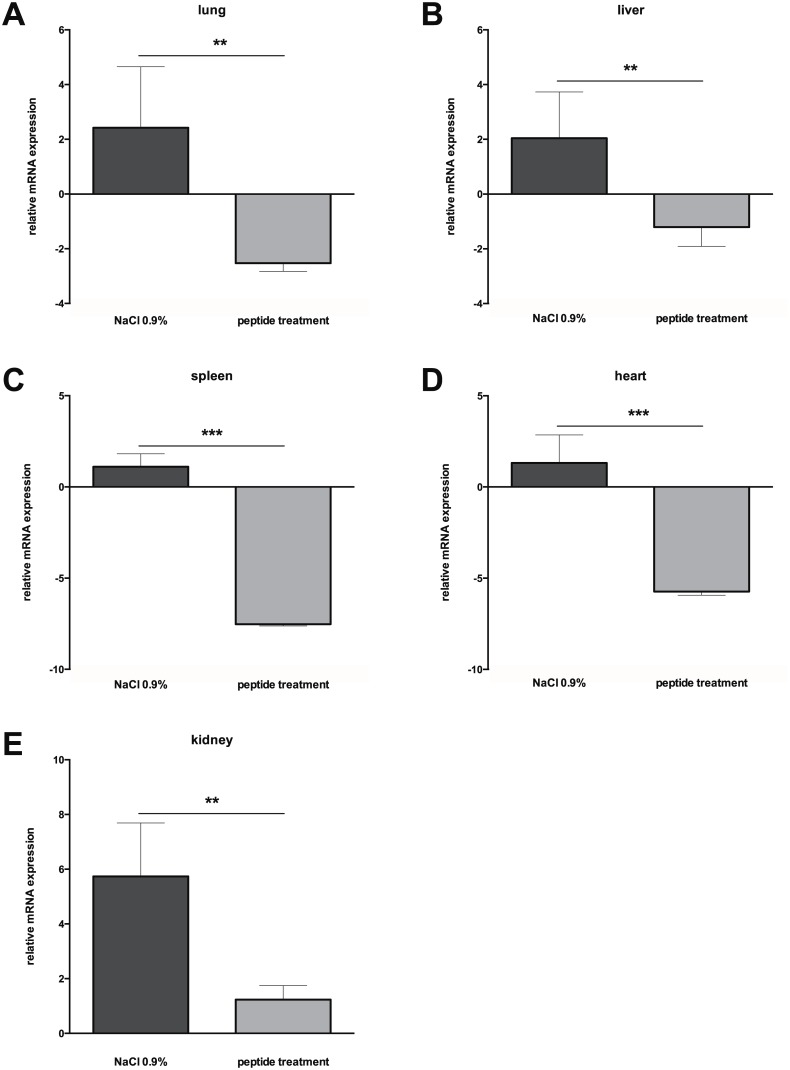
Relative mRNA expression of heparanase. Relative mRNA expression of heparanase were measured in lung (A), liver (B), spleen (C), heart (D) and kidney (E) of mice subjected to cecal ligature and puncture (CLP) and treated with peptide 19–2.5 (20 μg/ml, n = 6) or NaCl 0.9% as a control (n = 6). Data are presented as mean ± SD. P-values represent the statistical differences between groups using a multiple t-test with Holm-Šídák correction. **p < 0.01, ***p < 0.0001.

We induced sepsis in mice using cecal ligation and puncture (CLP), mimicking polymicrobial abdominal sepsis. To determine whether peptide 19–2.5 impacts on heparanase during sepsis *in vivo*, we treated septic mice with peptide 19–2.5 (peptide treatment) or saline (NaCl 0.9%) as control. CLP-mice without treatment demonstrated significantly higher levels of plasma heparanase compared to mice treated with peptide 19–2.5 (Fig 1A). Relative heparanase mRNA expressions in lung, liver, spleen, heart and kidney were significantly higher in untreated CLP-mice compared to mice treated with peptide 19–2.5 (Fig 2A–2E). However, we detected organ-specific differences. For example, heparanase mRNA expression was increased 5.7±1.9 fold in kidney (Fig 2E) but only 2.2±2.2 fold in lung (Fig 2A). The systemic response in sepsis encompasses both pro- and anti-inflammatory phases over time with an organ-specific time spread [22]. Notably, Schmidt et al. measured a peak in pulmonary heparanase expression 48h after CLP [20]. Thus, our results may be time-dependent and differ in a later stage of sepsis.

Peptide 19–2.5 decreased heparanase mRNA expression in all investigated organ tissues (Fig 2). Several studies report an up to 10-fold increase of heparanase expression after stimulation with pro-inflammatory cytokines [20,23–25]. In turn, we could demonstrate in previous experiments that peptide 19–2.5 decreases levels of pro-inflammatory cytokines in CLP-mice and decreases mRNA expression of CD14 [14]. The expression of CD14 showed differences between the organs in mice treated with peptide 19–2.5 [14]. Accordingly, we detected organ-specific differences of heparanase levels that may depend on cytokine levels and stage of sepsis.

To study heparanase levels in humans, we used plasma samples of septic shock patients (n = 18) and healthy human volunteers (n = 10). Levels of plasma heparanase were significantly higher in septic shock patients compared to healthy volunteers (Fig 1B). In both groups, the *ex vivo* addition of peptide 19–2.5 did not influence heparanase levels (Fig 1B). Recent studies reported elevated plasma heparanase levels in patients with diabetes mellitus and in pediatric patients with cancer. However, the reported levels are much lower compared to septic shock patients [26,27]. High levels of pro-inflammatory cytokines found in pathological conditions, such as sepsis, diabetes or cancer are the main inductors of heparanase expression [23]. Thus, higher levels of plasma heparanase in septic shock patients may be due to the higher levels of pro-inflammatory cytokines in sepsis [28].

### Peptide 19–2.5 reduces heparanase activity *in vivo and ex vivo*

Several studies identified elevated levels of circulating HS-fragments in critically ill patients [3,29,30] with significant higher levels in non-survivors [31]. Johnson et al. administered HS-fragments by intraperitoneal injection in mice resulting in eighty percent mortality. However, they used artificial amounts of 5 mg of HS-fragments for intraperitoneal injection [6]. Thus, attenuating the generation of circulating HS-fragments may serve as a crucial therapeutic effect in sepsis.

**Fig 3:**
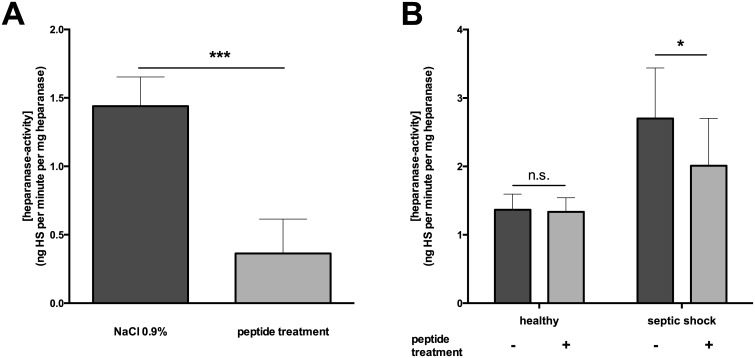
Heparanase activity in murine (A) and human (B) sepsis. Plasma was obtained from mice subjected to cecal ligature and puncture (CLP) and treated with peptide 19–2.5 (20 μg/ml, n = 6) or NaCl 0.9% as a control (n = 6). Furthermore, plasma samples were collected from control healthy volunteers (n = 10) and from patients with septic shock (n = 18). Peptide 19–2.5 (20 μg/ml) was added ex vivo to the plasma. Data are presented as mean ± SD. P-values represent the statistical differences between groups using a multiple t-test with Holm-Šídák correction. *p < 0.05, ***p < 0.0001, n.s. = non-significant.

Since heparanase is the major mammalian enzyme liberating circulating HS-fragments [5], we investigated heparanase activity in mice subjected to CLP. Treatment with peptide 19–2.5 resulted in significant lower heparanase activity compared to untreated control animals (Fig 3A). Schmidt et al. observed an association between LPS and activation of heparanase; LPS administration to mouse lung microvascular endothelial cells induced cleavage of 65-kDa heparanase to its active 50-kDa isoform. Additionally, the inhibition of heparanase is lung-protective in murine sepsis [20]. Recently, it was shown that peptide 19–2.5 changes the aggregate structure of LPS thereby neutralizing the pro-inflammatory effects of LPS [12]. Thus, peptide 19–2.5 may lead to a lower heparanase activity in polymicrobial sepsis by neutralizing LPS.

Next, we found significantly elevated heparanase activity in plasma samples from septic shock patients compared to healthy humans (Fig 3B). Notably, Schmidt et al. detected that plasma HS degradation activity is elevated only in individuals with non-pulmonary sepsis [20]. Although limited by a small sample size, our study included septic shock patients with pulmonary and non-pulmonary focus of infection (Table 1). The addition of peptide 19–2.5 to the plasma of healthy humans did not alter heparanase activity. However, peptide-addition to the plasma of septic shock patients significantly lowered heparanase activity (Fig 3B). These findings may be due to the significant lower levels of heparanase in healthy human plasma compared to plasma of septic shock patients. Probably, in samples with heparanase below a certain threshold, the accompanying levels of cytokines and HS are too low to allow a measurable effect of peptide 19–2.5.

**Fig 4:**
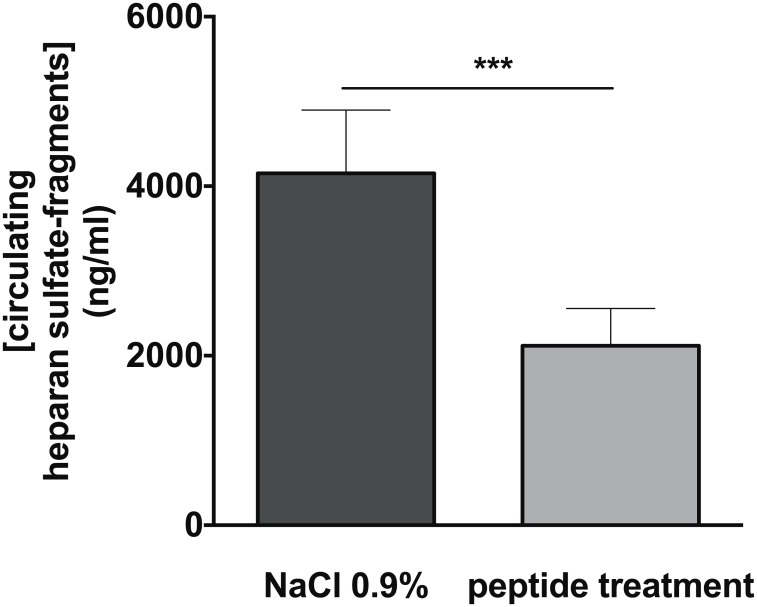
Levels of circulating heparan sulfate-fragments in murine sepsis. Levels of circulating heparan sulfate-fragments (HS-fragments) levels were measured in plasma of mice subjected to cecal ligature and puncture (CLP) and treated with Peptide 19–2.5 (20 μg/ml, n = 6) or NaCl 0.9% as a control (n = 6). Data are presented as mean ± SD. P-values represent the statistical differences between groups using a multiple t-test with Holm-Šídák correction. *** p < 0.0001.

Recently, we reported that peptide 19–2.5 is able to decrease the inflammatory response in murine cells stimulated with HS-fragments *in vitro* [3]. Thus, we investigated the impact of peptide 19–2.5 on levels of circulating HS-fragments *in vivo*. Levels of circulating HS-fragments significantly decreased in CLP-mice treated with peptide 19–2.5 compared to untreated control animals (Fig 4). We postulate two ways by which peptide 19–2.5 reduces levels of circulating HS-fragments in mice:

1. Interaction with heparanase and reduction of heparanase activity.
2. Direct binding between peptide and circulating HS-fragments.

Remarkably, Krepstakies et al. reported an alteration of the peptide’s secondary structure and a characteristic change in the hydration and sulfation status of HS after incubating with peptide 19–2.5 [13].

Of note, heparanase induces the release of pro-inflammatory cytokines through the generation of circulating HS-fragments [5], which in turn induce the expression of heparanase [23]. Thus, reducing the level of circulating HS-fragments by peptide 19–2.5 interrupts this cycle of pro-inflammatory action.

### Enthalpy changes of the interaction between peptide 19–2.5 and circulating heparan sulfate-fragments

**Fig 5:**
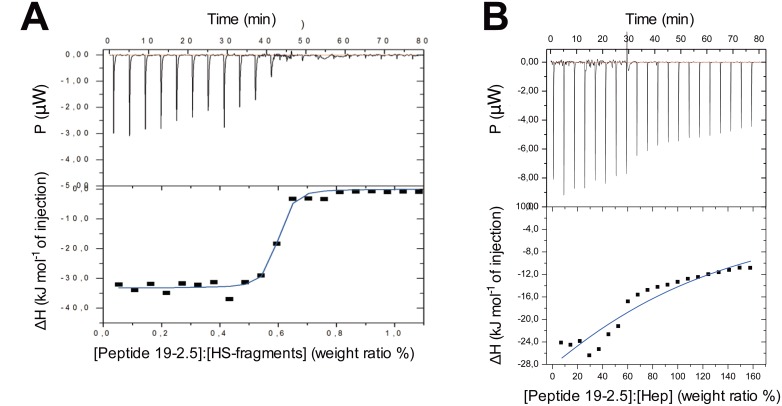
Isothermal titration calorimetry (ITC). For the ITC experiments, 2 mM peptide 19–2.5 was titrated to this dispersion stepwise in 3 μl portions into (A) 200 μg/ml heparan sulfate-fragments (HS) and (B) 100 μg/ml heparanase (Hep). In each titration, the resulting heat signals P were recorded (peak downwards are exothermic, peaks upwards endothermic reactions) against time (min) and presented in the top parts of Fig 5A and 5B. The integrated enthalpy changes ΔH versus [peptide]:[HS-fragments] (A) and [peptide]:[heparanase] (B) concentration ratios of the single titrations are illustrated in the bottom parts of Fig 5A and 5B.

To study the binding of peptide 19–2.5 with HS-fragments and heparanase we performed isothermal titration calorimetry (ITC). This technique allows statements about the kind of binding, such as Coulomb interactions leading to exothermic reactions or entropy-governed processes such as dissociation of water layers leading to endothermic reactions. First, peptide 19–2.5 was titrated to HS-fragments. There was a high exothermic reaction due to the single titrations which runs into saturation around a [Peptide 19–2.5]:[HS] weight ratio of 0.6 (Fig 5A). This indicates a strong Coulomb interaction between peptide 19–2.5 and HS. The thermodynamic parameters deduced from such experiments (n = 4) are summarized in Table 2.

**Table 2:** Thermodynamic parameters of the peptide 19–2.5 binding to HS-fragments.

Number of experiment	N (w/w ratio)	K_a_ (M^-1^)	K_d_ (M)	ΔS (cal/mol/K)	ΔG (cal/mol)
1	0.511	1.93×10^6^	5.18x10^-7^	4.46	-8918
2	0.475	1.52×10^7^	6.57x10^-8^	6.42	-10192
3	0.547	1.55×10^7^	6.45x10^-8^	10.8	-10201
4	0.534	1.20×10^7^	8.33x10^-8^	6.77	-10046
Mean	0.520	1.12×10^7^	8.92x10^-8^	7.11	-9839
SD	0.032	6.35×10^6^	1.57x10^-7^	2.66	618

N stoichiometry of binding, K_a_ binding constant, ΔH enthalpy change, ΔS entropy change, ΔG Gibbs free energy, SD standard deviation.

The interaction between peptide 19–2.5 and HS-fragments seems to result from a Coulomb binding of the positive peptide charges with the negatively charged sulfate groups from HS-fragments, and the resulting S-shaped curve with saturation characteristic for a complex formation of the peptide with HS-fragments (Fig 5A). These results are in line with the findings from Krepstakies et al. who investigated the impact of peptide 19–2.5 on virus attachment and entry of human pathogenic viruses by interacting with heparan sulfate proteoglycan [13]. A similar procedure was followed to determine the thermodynamic parameters of binding of peptide 19–2.5 with HS-fragments, however 1 μl of peptide was used for every titration [13].

It is important to note, that the high binding constant (Table 2) for this interaction dramatically decreases when the hydrophobic C-terminal of the peptide, FWFWG, is removed from the peptide (peptide variant peptide 19–2.5gek GCKKYRRFRWKFKGK). Thus, beside a Coulomb interaction a second binding process must take place, which is governed apparently by hydrophobic interactions.

### Enthalpy changes of the interaction between peptide 19–2.5 and heparanase

Next, we investigated the interaction of peptide 19–2.5 with heparanase (Fig 5B). ITC revealed an exothermic reaction when peptide 19–2.5 was titrated to heparanase, corresponding to negative enthalpy changes (ΔH) of approximately −25 kJ/mol at the beginning of the experiment. Although an exothermic reaction can be observed between peptide 19–2.5 and heparanase (Fig 5B), there are considerable differences from those described for HS-fragments (Fig 5A): First, the ΔH values at the beginning of the titration are much lower than those for the HS-fragments titration. Second, they do not have a saturation character, because there is no sigmoidal curve and there is still an essential ΔH value constantly around 12 kJ/mole at high peptide concentrations. This behavior does not allow calculating a binding constant as in the case of HS-fragments. Third, the processes, which take place, are at peptide concentrations being orders of magnitude higher than those for the interaction with HS-fragments.

A polar interaction should take place by binding of the positive peptide charges with the acidic (negative) charges of the protein, although the latter has more positive (100) than negative (86) charges. This indicates that there are no clear binding sites for the peptide in heparanase, and the interaction seems to have a more charge-independent, at least partially hydrophobic character. Taken together, for the high affinity binding of peptide 19–2.5 with HS-fragments as well as heparanase a two-step mechanism takes place. A direct Coulomb interaction between the positive charges of the peptide and the negative groups of HS-fragments or heparanase takes place as initial step. In a second step a hydrophobic interaction between the respective partners takes place. This leads to an activity decrease of the enzyme heparanase as well as a binding and characteristic change in the hydration and sulfation status of HS-fragments. Most importantly, however, the conformational change of the heparanase induced by the peptide causes a significant decrease of its enzymatic activity (Fig 3A and 3B).

## Conclusions

In summary, our data indicate for the first time that plasma heparanase level and activity are elevated in murine and human sepsis. Moreover, we demonstrated that the synthetic antimicrobial peptide 19–2.5 interacts with heparanase and consecutively attenuates the liberation of circulating HS-fragments in systemic inflammation. The recently reported interaction between peptide 19–2.5 and PAMPs *in vitro* [3] is confirmed by our current study *in vivo*. Thus, peptide 19–2.5 may be a potential anti-inflammatory agent in sepsis by interacting with heparanase and circulating HS-fragments.
